# Effect of large weight reductions on measured and estimated kidney function

**DOI:** 10.1186/s12882-017-0474-0

**Published:** 2017-02-06

**Authors:** Bernt Johan von Scholten, Frederik Persson, Maria S. Svane, Tine W. Hansen, Sten Madsbad, Peter Rossing

**Affiliations:** 1 Steno Diabetes Center Copenhagen, Niels Steensens Vej 1, 2820 Gentofte, Denmark; 2 Hvidovre University Hospital, Hvidovre, Denmark; 3 University of Copenhagen, Copenhagen, Denmark; 4 Aarhus University, Aarhus, Denmark

**Keywords:** Glomerular filtration rate, Bariatric surgery, Creatinine, Muscle mass, Cystatin C, DXA scan

## Abstract

**Background:**

When patients experience large weight loss, muscle mass may be affected followed by changes in plasma creatinine (pCr). The MDRD and CKD-EPI equations for estimated GFR (eGFR) include pCr. We hypothesised that a large weight loss reduces muscle mass and pCr causing increase in eGFR (creatinine-based equations), whereas measured GFR (mGFR) and cystatin C-based eGFR would be unaffected if adjusted for body surface area.

**Methods:**

Prospective, intervention study including 19 patients. All attended a baseline visit before gastric bypass surgery followed by a visit six months post-surgery. mGFR was assessed during four hours plasma ^51^Cr-EDTA clearance. GFR was estimated by four equations (MDRD, CKD-EPI-pCr, CKD-EPI-cysC and CKD-EPI-pCr-cysC).

DXA-scans were performed at baseline and six months post-surgery to measure changes in lean limb mass, as a surrogate for muscle mass.

**Results:**

Patients were (mean ± SD) 40.0 ± 9.3 years, 14 (74%) were female and 5 (26%) had type 2 diabetes, baseline weight was 128 ± 19 kg, body mass index 41 ± 6 kg/m2 and absolute mGFR 122 ± 24 ml/min. Six months post-surgery weight loss was 27 (95% CI: 23; 30) kg, mGFR decreased by 9 (−17; −2) from 122 ± 24 to 113 ± 21 ml/min (*p* = 0.024), but corrected for current body surface area (BSA) mGFR was unchanged by 2 (−5; 9) ml/min/1.73 m^2^ (*p* = 0.52). CKD-EPI-pCr increased by 12 (6; 17) and MDRD by 13 (8; 18) (*p* < 0.001 for both), while CKD-EPI-cysC was unchanged by 2 (−8; 4) ml/min/1.73 m^2^ (*p* = 0.51). Lean limb mass was reduced by 3.5 (−4.4;−2.6; *p* < 0.001) kg and change in lean limb mass correlated with change in plasma creatinine (*R* ^2^ = 0.28, *p* = 0.032).

**Conclusions:**

Major weight reductions are associated with a reduction in absolute mGFR, which may reflect resolution of glomerular hyperfiltration, while mGFR adjusted for body surface area was unchanged. Estimates of GFR based on creatinine overestimate renal function likely due to changes in muscle mass, whereas cystatin C based estimates are unaffected.

**Trial registration:**

ClinicalTrials.gov, NCT02138565. Date of registration: March 24, 2014.

## Background

Accurate assessment of glomerular filtration rate (GFR) is important, both to evaluate the progression of renal disease, and to monitor the effect of intervention on kidney function as well as to inform drug dosing and patient counselling. However, optimal methods of measuring kidney function in the setting of obesity or longitudinally in the setting of weight change are uncertain.

Plasma creatinine is frequently used to estimate GFR, since it has proven to be an inexpensive and reliable index of kidney function. The primary determinant of creatinine generation/production is skeletal muscle mass where the final catabolite of muscular energetic metabolism is creatinine [1]. Hence, if body weight - and muscle mass in particular - changes over time and leads to changes in plasma creatinine, this may affect estimates of kidney function, without actual changes in accurately measured GFR. Whether these factors impact eGFR could depend on the equations applied, as the 4-variable Modification of Diet in Renal Disease (MDRD) [2] and Chronic Kidney Disease Epidemiology Collaboration (CKD-EPI) [3] equations include plasma creatinine and would not be affected body weight changes alone. Cystatin C is a filtration marker that is less influenced by changes in muscle mass and may be a more suitable marker of renal function in subjects experiencing fast and large weight reductions [4]. In this prospective intervention study, we investigated the effect of a large weight loss (after Roux-en-Y gastric bypass surgery (RYGB)) on measured GFR (mGFR) (^51^Cr-EDTA plasma clearance) and on estimated GFR (using both plasma creatinine and cystatin C). Dual energy X-ray absorptiometry (DXA)-scans were performed before and after RYGB to estimate changes in skeletal muscle mass.

We hypothesised that a large weight loss reduces muscle mass (lean limb mass) and plasma creatinine leading to increases in eGFR (creatinine-based equations), whereas mGFR and cystatin C-based eGFR would be unaffected when adjusted for the change in body surface area (BSA).

## Methods

### Participants and study design

This prospective, open-label intervention study included 23 obese patients all scheduled for RYGB at Hvidovre University Hospital, Denmark. Three patients never had the surgery performed and one patient declined to participate in the post-surgery visit. Therefore, a total of 19 patients completed the study. Type 2 diabetes was diagnosed according to the WHO criteria.

Patients were recruited from March 2014 and the study was completed in June 2016.

**Fig. 1:**
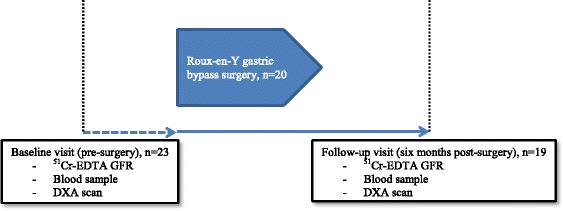
Study design

The study design is illustrated in Fig. 1. Patients attended the baseline visit within two weeks prior to the scheduled RYGB and the follow-up visit was performed six months (±2 weeks) after RYGB.

The study protocol was approved by the regional ethics committee and was conducted according to the Declaration of Helsinki. All patients gave written informed consent before any study procedure was initiated. The study is registered at ClinicalTrials.gov with identifier NCT02138565. The two study-related visits were conducted at Steno Diabetes Center, Gentofte, Denmark, and the RYGB was performed at Hvidovre University Hospital, Hvidovre, Denmark as described previously [5].

The primary aim was to assess the effect of a large weight reduction on measured GFR (^51^Cr-EDTA plasma clearance) and on estimated GFR (applying different equations based on plasma creatinine or cystatin C). Secondly, we assessed the effect on body composition (determined by DXA-scan) in order to relate these changes with changes in renal outcome measures.

### Outcome measurements

Renal function (mGFR) and extracellular volume were assessed during four hours measurement of plasma ^51^Cr-EDTA clearance by standard methods [6]. ^51^Cr-EDTA was performed within two weeks prior to RYGB and six months (± two weeks) after surgery.

For the present study, mGFR was represented by two approaches: 1) Absolute mGFR, where mGFR was presented as the raw mGFR (ml/min) and 2) mGFR corrected for concurrent body surface area (BSA): BSA-corrected mGFR (ml/min/1.73 m^2^).

For the estimation of BSA we used the Du Bois & Du Bois formula [7].

IDMS-traceable plasma creatinine was analysed using the enzymatic Creatinine Plus method (Vitros 5600, Ortho Clinical Diagnostics, Illkirch Cedex, France). Standardized plasma cystatin C was analyzed on the Cobas 8000® (Roche Diagnostics, Indianapolis, IN).

Estimated GFR was calculated by four equations:

1) Creatinine equation (MDRD 1999): MDRD eGFR [2]; 2) Creatinine equation (CKD-EPI 2009): CKD-EPI-pCr eGFR [3]; 3) Cystatin C equation (CKD-EPI 2012): CKD-EPI-cysC eGFR [8]; and 4) Creatinine-cystatin C equation (CKD-EPI 2012): CKD-EPI-pCr-cysC eGFR [8].

DXA measurements of body composition were performed in all patients at baseline and six months after surgery using a Hologic Discovery A, series 82800-A (Hologic, Bedford, MA, USA).

The following parameters were obtained: Lean body mass (in kg), lean limb mass (in kg), fat mass (in kg) and fat mass (in percent). Lean limb mass is considered the best surrogate measure of skeletal muscle mass and was calculated as the total non-bone and non-fat lean mass of the extremities: Lean mass of left arm + lean mass of right arm + lean mass left leg + lean mass of right leg [9].

Urinary albumin-to-creatinine ratio (UACR) was calculated as the geometric mean of three consecutive morning spot urine samples performed at baseline and six months after surgery.

Twenty-four-hour blood pressure was performed at baseline and six months after surgery using BPro (HealthStats, Singapore), a watch-like device that captures radial pulse wave reflection and calculates brachial 24–h BP. BPro has been validated in people with diabetes and meets the European Society of Hypertension and Association for the Advancement of Medical Instrumentation standards [10, 11]. Mean of systolic and diastolic blood pressure and heart rate was calculated using all readings during the 24 h. Only 24-h blood pressure recordings with ≥ 20 readings during daytime and ≥ 7 during night-time were used for analysis. One recording was incomplete and was discarded for the analysis.

The urinary albumin concentration of the morning spot samples was analysed using a turbidimetric immunoassay (Vitros 5600, Ortho Clinical Diagnostics, Illkirch Cedex, France).

### Statistical analysis

Outcome measures are presented as mean (SD) and skewed data (UACR) are shown as geometric mean (IQR), and analysed after log-transformation.

The change in outcome measures was analysed from levels at baseline to six months after surgery and compared using the paired samples t-tests.

Associations between changes in outcome measures were assessed by linear regression models. The proportion of the variability explained by the models is presented as the *R* ^2^. Due to the exploratory nature of the study, no power calculation was performed, however based on a previous related study, we anticipated that a total of 20 subjects would be sufficient [12].

Two-sided *p*-values < 0.05 were considered statistical significant. Statistical analysis was performed using IBM SPSS 23.0 (IBM Amonk NY, USA).

## Results

### Baseline demographics

Patients were (mean ± SD) 40 ± 9 years, 14 (74%) were female and 5 (26%) had type 2 diabetes. Baseline weight was 128 ± 19 kg, body mass index 41 ± 6 kg/m^2^, absolute mGFR 122 ± 24 ml/min and CKD-EPI-pCr eGFR 93 ± 18 ml/min/1.73 m^2^. Six patients received antihypertensive treatment at baseline and no changes were prescribed during the course of the study.

### Renal outcome measures

Six months after RYGB, absolute GFR was reduced by mean 9 (95% confidence interval: 2; 17; *p* = 0.021) ml/min, while BSA-corrected GFR was unchanged by 2 (−5; 9; *p* = 0.52) ml/min/1.73 m^2^ (Table 1).

**Table 1:** Renal outcome measures

Variable	Baseline	6 months post-surgery	Change from baseline (95% CI)	*p*-value
mGFR (^51^Cr-EDTA), ml/min (absolute)	122 (24)	113 (21)	−9 (−17; −2)	*0.021*
mGFR (^51^Cr-EDTA), ml/min/1.73 m^2^ (corrected for body surface area)	88 (17)	90 (16)	2 (−5; 9)	0.52
Body surface area, m^2^	2.38 (0.22)	2.14 (0.23)	0.24 (0.20; 0.28)	*<0.001*
Plasma creatinine, μmol/l	76 (18)	66 (12)	−9 (−14; −5)	*<0.001*
Plasma cystatin C, mg/l	0.94 (0.19)	0.96 (0.19)	0.02 (−0.04; 0.07)	0.61
CKD-EPI-sCr eGFR, ml/min/1.73 m^2^	93 (18)	105 (15)	12 (6; 17)	*<0.001*
MDRD eGFR, ml/min/1.73 m^2^	84 (21)	97 (22)	13 (8; 18)	*<0.001*
CKD-EPI-cysC eGFR, ml/min/1.73 m^2^	89 (19)	87 (23)	−2 (−8; 4)	0.51
CKD-EPI-sCr-cysC eGFR, ml/min/1.73 m^2^	91 (19)	96 (21)	5 (−3; 10)	0.074
24-h systolic blood pressure, mmHg	122 (14)	124 (13)	2 (−5; 10)	0.56
24-h diastolic blood pressure, mmHg	82 (10)	79 (11)	−3 (−9; 4)	0.41
Urinary albumin-to-creatinine ratio, mg/g	6.3 (2.7–8.1)	4.8 (2.1–5.2)	−23 (−35; −9) %	*0.005*
Extracellular volume, l	20.4 (5.9)	20.4 (6.3)	0 (−2.5; 2.6)	0.99
Plasma urea, mmol/l	5.0 (1.5)	4.3 (1.1)	−0.7 (−1.3; −0.02)	*0.043*
Plasma calcium, mmol/l	1.28 (0.04)	1.28 (0.03)	0 (−0.02; 0.02)	0.74

Values represent mean (SD) or geometric mean (IQR)

*GFR* glomerular filtration rate

Plasma creatinine was reduced by 9 (5; 14; *p* < 0.001) μmol/l, and plasma cystatin C was unchanged by 0.02 (−0.04; 0.07; *p* = 0.61) six months after RYGB. MDRD eGFR increased by 13 (8; 18; *p* < 0.001) ml/min/1.73 m^2^, CKD-EPI-pCr eGFR increased by 12 (6; 17; *p* < 0.001) ml/min/1.73 m^2^, and CKD-EPI-pCr-cysC eGFR was unchanged by 5 (−0.5; 10; *p* = 0.074) ml/min/1.73 m^2^. CKD-EPI-cysC eGFR was unchanged by 2 (−8; 4; *p* = 0.51) ml/min/1.73 m^2^. Plasma urea was reduced by 0.7 (−1.3; −0.02; *p* = 0.043) mmol/l and UACR was reduced by 23 (−35; −9; *p* = 0.005) %, while extracellular volume was unchanged (*p* = 0.99) (Table 1).

### Weight loss and body composition outcome measures

Six months after RYGB weight was reduced by mean 27 (23; 30; *p* < 0.001) kg or 21 (18; 24; *p* < 0.001) % and body mass index was reduced by 8 (−10; −7; *p* < 0.001) kg/m^2^. Lean limb mass was reduced by 3.5 kg (−4.4; −2.6; *p* < 0.001) kg, lean body mass was reduced by 6.5 (−7.9; −5.0; *p* < 0.001) kg, and fat mass was reduced by 20 (−23; −18; *p* < 0.001) kg (Table 2).

**Table 2:** DXA outcome measures

Variable	Baseline	6 months post-surgery	Change from baseline (95% CI)	*p*-value
Lean body mass, kg	66.2 (12.2)	59.7 (13.0)	−6.5 (−7.9; −5.0)	<0.001
Lean body mass + bone mineral content, kg	69.1 (12.4)	62.6 (13.3)	−6.5 (−7.9; −5.0)	<0.001
Lean limb mass, kg	30.6 (6.5)	27.1 (6.6)	−3.5 (−4.4; −2.6)	<0.001
Fat mass, kg	59.0 (12.0)	38.5 (9.9)	−20.4 (−23.1; −17.7)	<0.001
Fat mass, %	46.1 (5.8)	38.1 (6.2)	−7.9 (−9.1; −6.7)	<0.001
Weight, kg	128 (19)	101 (18)	−27 (−30; −23)	<0.001

Values represent mean (SD)

### Linear correlations

At baseline, BSA-corrected mGFR correlated significantly with plasma creatinine and with all estimates of GFR (*R* ^2^ ≥ 0.25, *p* ≤ 0.029), except for MDRD (*p* = 0.093). After RYGB, BSA-corrected mGFR correlated with plasma creatinine and all estimates of GFR (*R* ^2^ ≥ 0.34, *p* ≤ 0.011). Change in BSA-corrected mGFR correlated with change in plasma creatinine and MDRD eGFR (*R* ^2^ = 0.24, *p* ≤ 0.048) and not with changes in other renal measures.

Lean limb mass correlated significantly with plasma creatinine at baseline (*R* ^2^ = 0.28, *p* = 0.025) and after RYGB (*R* ^2^ = 0.37, *p* = 0.010). Change in lean limb mass correlated with change in plasma creatinine (*R* ^2^ = 0.28, *p* = 0.032) and with change in UACR (*R* ^2^ = 0.28, *p* = 0.034).

Lean body mass correlated significantly with plasma creatinine at baseline (*R* ^2^ = 0.32, *p* = 0.012) and after RYGB (*R* ^2^ = 0.42, *p* = 0.004). Change in lean body mass correlated with change in UACR (*R* ^2^ = 0.38, *p* = 0.011), and not with change in plasma creatinine or other renal measures (*p* ≥ 0.38).

## Discussion

In this prospective intervention study investigating the effects of a fast and large (mean 27 kg) weight loss, obtained by Roux-en-Y gastric bypass surgery, we found a reduction in absolute mGFR, while BSA-corrected mGFR was unchanged. Plasma creatinine was reduced causing increases in creatinine-based eGFR (MDRD and CKD-EPI), while cystatin C-based eGFR was unchanged (all adjusted for BSA). Lean limb mass, a surrogate measure of skeletal muscle mass, was reduced by mean 3.5 kg and might explain the reduction in plasma creatinine, since we found a significant correlation between these changes.

Monitoring GFR is important for diagnosis and monitoring of patients with kidney disease. Furthermore, it is often used for dosage of drugs, mainly for safety reasons. Numerous equations based on plasma creatinine have been suggested for estimation of GFR and when compared to accurately measured GFR, particularly MDRD and CKD-EPI have been proven to be reliable. Of note, creatinine-based eGFR equations have not been validated in morbidly obese adults or in patients with change in body composition after RYGB [13]. Cystatin C is less affected by muscle mass and diet than is creatinine, while reports have found an association between cystatin C concentrations and body weight and fat mass [14–17]. In our study, cystatin C levels tended to be associated with body weight, but were not associated with fat mass, fat percent or body mass index (data not shown). Nonetheless, it has been anticipated that cystatin C would provide a more accurate estimate of GFR than creatinine [18]. Measuring GFR by inulin-clearance [19], chromium-EDTA clearance [6] or iohexol clearance [20] is considered the “gold standard” of GFR. However, it is expensive and time consuming (usually a four hour examination), and therefore not realistic as a routine measurement in clinical practice or in large-scale studies. Whether mGFR should be presented absolute or BSA-corrected (expressed as per 1.73 m^2^) in the setting of obesity is still unclear and may depend on the situation [13]. However, studies have indeed questioned the use of BSA-correction and concluded that data obtained for GFR indexed by BSA should either be avoided or interpreted with caution, especially in obese subjects [21, 22]. Due to the limitations of BSA-correction, the extracellular volume has been proposed as a better parameter for body size adjustment of GFR than BSA [23]. In our study, the extracellular volume was essentially unchanged six months after RYGB, hence mGFR adjusted for extracellular volume was reduced. However GFR indexed with extracellular volume is currently not recommended [24].

Taken together, the optimal methods of measuring kidney function in obese subjects and after weight changes are debated but still indeterminate. The purpose of the present study was to obtain a better understanding of how a large weight loss influence measured and estimated kidney function. The study hypothesis was that change in creatinine-based eGFR would be different than change in mGFR, since a large weight reduction would lead to a reduction in muscle mass affecting plasma creatinine levels without impacting BSA-corrected mGFR. Our primary findings were that absolute mGFR was reduced, BSA-corrected mGFR and cystatin C-based eGFR were unchanged, while creatinine-based eGFR was increased after a weight reduction of mean 27 kg. By applying robust methods for determination of body composition, we were able to demonstrate that changes in muscle mass correlated with changes in plasma creatinine. This suggests that for monitoring changes in renal function over time in patients experiencing a large weight loss, cystatin C-based estimates of GFR may be more useful.

Other studies have demonstrated similar results. In a study of 37 patients, a weight loss of 37 kg six months after surgery was associated with a significant reduction in mean creatinine, and accordingly an increase in MDRD, while Cockcroft Gault eGFR (including both creatinine and body weight) was decreased [25]. A small prospective study demonstrated that BSA-corrected mGFR, determined using clearance of iothalamate, was reduced in a cohort of 11 women during the first year after bariatric surgery. Notably, serum creatinine and creatinine-based eGFR did not identify this change in renal function, which was explained by a large reduction in creatinine production. Preoperatively, the CKD-EPI equation underestimated mGFR; postoperatively, mGFR was overestimated due to the reduction in body weight and muscle mass [12]. Of note, these studies did not measure actual changes in body composition. In a recent pooled analysis including more than 5000 patients, we assessed whether a pharmaceutically induced weight loss was associated with changes in plasma creatinine. We demonstrated that a “stable” weight reduction of mean 1.9 kg was not associated with a change in plasma creatinine and concluded, that in patients experiencing a smaller weight reduction, creatinine-based equations (MDRD and CKD-EPI) are unaffected and can be applied [26]. The extent and rate of a weight reduction associated with enough impact on skeletal muscle mass reduction to affect levels of creatinine and eGFR is currently unknown and cannot be determined by the present study. Depending on the magnitude of the weight reductions, a non-creatinine-based equation (e.g. cystatin C) should be considered for these studies, in order to obtain reliable estimates of kidney function.

Our present study expands on previous studies investigating the effects of bariatric surgery on mGFR. In studies examining mGFR in patients with normal or supranormal kidney function, absolute mGFR decreased significantly, while the BSA-corrected mGFR was unchanged one year after surgery [13, 27]. In the present study, we can confirm these findings and in a sub-analysis of subjects with hyperfiltration (baseline mGFR > 130 ml/min, *n* = 5) mGFR was significantly reduced by 24 ml/min (data not shown). This illustrates that the GFR-lowering effect of bariatric surgery is more pronounced in subjects with supranormal baseline levels of mGFR. It has been suggested that the decrease in the absolute mGFR is a resolution of glomerular hyperfiltration which may result in decreased intraglomerular pressure and kidney injury [13, 27, 28].

In a recent study, including 985 patients treated with bariatric surgery and 985 matched controls, it was concluded that patients undergoing bariatric surgery had a 58% lower risk of an eGFR decline ≥ 30% and a 57% lower risk of doubling of serum creatinine or developing end-stage renal disease compared with the controls. Of note, end-stage renal disease occurred in only eight surgery and ten non-surgery patients, indicating that the vast majority of the kidney outcomes were based on levels of creatinine [29]. While the study was well-designed and provided valuable information with important clinical implications, a major limitation is the use of a creatinine-based eGFR for determination of kidney outcomes, as also highlighted by the authors themselves. In our study, plasma creatinine was reduced in all patients except two, likely explained by a reduction in muscle mass. We found absolute mGFR and creatinine-based eGFR to change in opposite directions after RYGB. In order to resolve whether bariatric surgery is reducing the risk of adverse kidney outcomes, cystatin C-based eGFR may be a more suitable measure.

### Strengths and limitations

Strengths of this study include the prospective design and the use of robust methods to determine changes in renal function and body composition after RYGB. Limitations include the small sample size and lack of a non-surgical control group with a comparable weight reduction. As a result, we are unable to rule out that our findings are not specifically caused by the RYGB-related changes in the renal outcome measures.

## Conclusions

Major weight reductions are associated with a reduction in absolute mGFR, which may reflect resolution of glomerular hyperfiltration, while mGFR adjusted for body surface area was unchanged. Estimates of GFR based on plasma creatinine overestimate renal function likely due to changes in muscle mass, whereas cystatin C based estimates are unaffected. Our results have important implications for both clinicians and researchers and provide a better understanding of the physiology of glomerular filtration rate and emphasize the limitations of using plasma creatinine in the setting of obesity and following weight changes.

## Abbreviations

BSA: Body surface area
CKD-EPI: Chronic Kidney Disease Epidemiology Collaboration
CysC: Cystatin C
DXA: Dual energy X-ray absorptiometry
eGFR: Estimated glomerular filtration rate
GFR: Glomerular filtration rate
IQR: Interquartile range
MDRD: Modification of Diet in Renal Disease
mGFR: Measured glomerular filtration rate
RYGB: Roux-en-Y gastric bypass surgery
SD: Standard deviation
UACR: Urinary albumin-to-creatinine ratio
